# Little evidence for community treatment orders – a battle fought with heavy weapons

**DOI:** 10.1192/pb.bp.115.052373

**Published:** 2016-06

**Authors:** Reinhard Heun, Subodh Dave, Paul Rowlands

**Affiliations:** 1 Derbyshire Healthcare NHS Foundation Trust, Derby, UK

## Abstract

This editorial discusses the pros and cons of community treatment orders (CTOs) from the perspective of community general adult psychiatry. There is little scientific evidence supporting the application of CTOs. Preconditions of a CTO to work are likely to be met by few patients. The time for the application of a CTO may be better spent for patient-centred care until there is sufficient new and robust evidence that identifies the patients that might profit.

The legislative framework for treatment of mental disorder has evolved in the UK over more than a hundred years, with the successive acts of 1890, 1930, 1959 consolidating and refining the preceding common-law and statutory acts into a framework that became the Mental Health Act 1983. Community treatment orders (CTOs) were implemented in the 2007 Mental Health Bill amending the Mental Health Act.

The discussion about CTOs seems to be ongoing and is fought on both sides with heavy weapons – science, personal experience and best intentions. Sadly, there seems to be no resolution or agreement in sight among patients, carers and professionals.^1,2^ Supporters argue that we must help unwilling patients to take their medication and accept treatment in their own best interest or in the interest of others around them, even if it is with coercion under a CTO; uncontrolled real-life trials, anecdotal personal evidence and CTO use in 70 jurisdictions^3^ have suggested that CTOs may work.^4^ In contrast, opponents aver that all three randomised controlled CTO trials and their meta-analysis have shown absolutely no evidence that CTOs have any significant effect on the treatment outcome of patients.^5^

This editorial will provide an overview of the arguments on both sides. We will also focus on the practical side of implementation within the current legal framework: what are the preconditions for a CTO to do what it is intended to do, that is reduce hospital admissions and improve outcomes? These practical issues may not have been fully assessed in previous discussions about the CTO. We will refer to the literature and contextualise this to our clinical experience as general psychiatrists with several years' experience of working with CTOs within community services. We will end with some specific recommendations.

## The arguments on the pro-side

Supporters of CTOs put forward a number of arguments in their favour, some of which are listed below.

- Coercion with treatment for mental disorder in the patients' best interest is justified on ethical grounds and is a feature of the legislative arrangements in many jurisdictions.^6^
- There is no clear logical reason why this right or duty to appropriate treatment should be available in the restrictive hospital setting, but not in the community.
- For this purpose, it can be necessary to force non-adherent patients to accept necessary treatment and medication.^4^
- There is sufficient professional experience of patients improving under a CTO to justify this type of coercion.^4^
- Many clinical studies of CTOs have shown relevant benefits for patients.^7–10^
- Randomised controlled trials (RCTs) with negative results have not included the right group of patients that are likely to benefit from a CTO^4^ or have not applied the CTO appropriately.^11^

## The arguments on the con-side

Counter-arguments are being proposed by those who believe CTOs are not as beneficial to patient care as their supporters assert. We have collected some of those arguments below.

- Many patients relapse under a CTO.^12^
- CTOs increased health service use.^13^
- A number of non-randomised studies have provided negative and conflicting results and thus have not provided sufficient evidence to support CTOs.^3,14,15^
- Three independent RCTs and a meta-analysis of their data have shown no benefit of the CTO on the number of hospital admissions and other relevant outcomes.^16–20^
- Patients on a CTO have shown even less adherence to depot injections than those not on a CTO.^21^
- Anecdotal reports do not provide significant evidence for the efficacy of a CTO. They may be the result of the regression to the mean (i.e. the CTO is implemented at a time when the patient is at their most ill) and improvements are therefore the likely results of natural variance of the disease course.^22^
- Patients' human rights might be violated by CTOs.^23^
- Owing to flaws in the application of compulsory community care, patients are at risk of being subjected to new forms of social control of an unclear nature without proper legal protection.^24^
- Without evidence there cannot be any ethical justification to use coercion and severe deprivation of freedom and liberty against psychiatric patients.^3^

## Necessary conditions for a CTO to work in principle

Psychiatrists in the National Health Service (NHS) work within a legislative framework that includes CTOs. Community psychiatrists with typically sized case-loads will inevitably have experience of working with patients who are subject to CTOs and will have experienced the ethical dilemmas they present. Assuming that CTOs are effective in at least some individual patients, there are necessary preconditions which must be fulfilled for the order to work.

- The patient has a treatable mental disorder (i.e. a disorder that has shown some response to treatment).
- The patient does not want to continue to take the medication that is likely, from the perspective of the treating psychiatrist, to help maintain improvement and reduce risk of relapse. This may be for a range of reasons including side-effect burden, a disagreement that the medication is responsible for any improvement, a subjective perception that the medication has not helped, a belief that the medication is not necessary to maintain wellness or a disagreement that the problem being treated is a treatable mental disorder.
- The subjective, implicit or explicit, benefit–disadvantage evaluation of the patient has thus led to a decision against the treatment continuing in the community.
- Previous treatment in the hospital has not been sufficiently effective to lead to remission or improvement and has not increased the insight of the patient into their condition and their willingness to accept treatment.
- The patient is fully informed about the CTO and understands the conditions of the CTO.^2^
- They then accept and submit to the conditions of the CTO.^2^
- The patient's experience of a hospital stay was negative and the possibility of a hospital readmission is seen as a sufficiently coercive or aversive threat.
- This threat is sufficient enough to make the patient change their previous rejection and to accept treatment they otherwise would not accept.
- This treatment then improves the patient's mental health and reduces the likelihood of admission to hospital.

The number of patients for whom all these conditions apply may be limited.

## Some practical points to consider

There are additional, very relevant issues, including some practical points to consider which we draw from our previous experience with CTOs in the UK since 2008.

- The administration of a CTO is time consuming, bureaucratic and draws time away from appropriate patient-centred care. (In our experience, mental health tribunal reports, manager's report, capacity assessments and CTO renewal assessment can take up to 8–20 h, depending on the patient.)
- A doctor writing a report or completing the necessary forms does not provide any direct, if any, therapeutic benefit for or influence on the day-to-day care of a patient.
- The increase in CTO use with the associated costly legal machinery of mental health tribunals has led to an increase in expenditure which diverts spending from direct patient care.
- Threats and coercion may negatively affect the patient–psychiatrist relationship. The applications of a CTO may support paternalistic practice,^25^ and thus have an impact on the psychiatrist's role as a patient supporter, therefore limiting their influence on the patient.
- Many patients do not know that they are under a CTO, are not fully informed or do not understand the regulations of a CTO.^26^
- CTOs are favoured by relatives and carers,^27^ possibly to force patients to comply with their wishes; this may on some occasions be in their own, but not necessarily in the patient's, best interest.
- The functional split between in-patient and out-patient consultants makes regulations and administration difficult. An in-patient consultant may utilise a CTO for one purpose, such as with a view to shorten an in-patient admission, without proper consultation with the psychiatrist and team who will have the task of implementing the order in the community over the longer term. In practice, it is our experience that collaborative planning of CTOs between in-patient and out-patient consultants is not a routine occurrence.^2^
- The power to recall a patient to hospital by one doctor alone may reduce clinical governance in comparison with a full Mental Health Act assessment. It may have the advantage of being implemented more easily than a full Mental Health Act assessment but this is potentially at the expense of a less complete and balanced assessment.
- The recall of a CTO does not allow treatment. Patients who are recalled may stay on a ward for up to 72 h just to wait for a Mental Health Act assessment to happen.
- The CTO may be used to submit a patient to less than optimal depot medication, and may prevent the search for better suitable alternative treatments.^28^
- It may be difficult to assume that the simple threat of a hospital admission actually reduces such admissions and our experience has been that this has not appeared to be sufficient. On the other hand, we also have anecdotal examples of patients who may have done well under the framework.

## Conclusions

The scientific evidence that CTOs work is weak at best.^3,10^ The likelihood that three independent controlled studies and their meta-analysis have led to false negative results is low. Under these circumstances, no clinical procedure would have any support from any regulatory institution. The use of coercion without or even against scientific evidence may be seen as unethical and might violate the patients' human rights. These circumstances may increase the stigma against psychiatry.^29^ However, it is impossible to disprove that CTOs may not work at an individual level in some patients. Those most likely to benefit would appear to be those where the above-mentioned individual conditions are all concurrently met. If any such cases had been included in these controlled studies, their positive outcome must have been mirrored by other patients with an equivalent negative outcome, namely more admissions and worse outcome.

Some of the conditions for a CTO to work are unfavourable: the initial subjective benefit of the treatment is low; the treatment in hospital has not been fully efficient to lead to remission and to sufficient insight; the hospital must be perceived as a sufficient threat to trigger better adherence in the community. This may increase the stigma against psychiatry.

## Recommendations

It could be argued that the time, money and resources spent on administration and report writing within the current CTO legislative framework would be better spent working with patients on developing more collaborative approaches to the ongoing management of their condition.

There may be nothing lost if the current CTO is replaced by the better governed use of Section 2 or 3 of the Mental Health Act, utilising the existing provisions of the Act, until the proponents of CTOs have, with some scientific rigour, identified the subgroup of patients for whom it may help and that it does what it is intended to do.

If there is a subset of patients who may benefit from this extension of coercion into the community, it is important that there is a better delineation of the group of patients who benefit through a proper scientific evaluation that does not rely on anecdotal evidence. Such studies are urgently needed to justify the continued application of CTOs in the UK and also worldwide^1,30,31^ within mental health systems that are continually under resource pressures.

It may also be helpful to look at how other countries, which have or do not have a comparable legislation, deal with this subgroup of non-adherent patients^23^ and indeed what other paths the UK could have taken in 2007.

We believe also that there is an urgent need for greater transparency over the resource costs associated with the system that has developed over the past 7 years and a debate over how such sums of money are best spent for the benefit of patients.
